# The evaluation of accuracy of serial rapid HIV test algorithm in the diagnosis of HIV antibodies among pregnant women in south east Nigeria

**DOI:** 10.1186/s13104-015-1454-8

**Published:** 2015-10-12

**Authors:** Ikechukwu Innocent Mbachu, Gerald Udigwe, Ikechebelu Joseph, Okonkwo John, Umeononihu Osita Samuel, Ugboaja Joseph, Mbachu Chioma Ngozi

**Affiliations:** Department of Obstetrics and Gynaecology, Nnamdi Azikiwe University Teaching Hospital, PMB 5025, Nnewi, Anambra State Nigeria; Department of Paediatrics, Nnamdi Azikiwe University Teaching Hospital, PMB 5025, Nnewi, Anambra State Nigeria

**Keywords:** Rapid HIV test, Serial algorithm, Accuracy, Pregnant women

## Abstract

**Background:**

Accurate HIV testing in pregnancy is critical to the prevention of mother to child transmission of HIV infection and linkages to other preventive strategies.

**Aims and objectives:**

This study determined the sensitivity, specificity negative and positive predictive value of serial rapid testing of HIV among pregnant women in Nnewi, south east Nigeria.

**Methodology:**

This was a comparative descriptive study conducted over a 4-month period. Serial rapid testing algorithm was compared with conventional ELISA testing after obtaining informed consents from the pregnant women. All positive and discordant results were confirmed with western blot HIV test. Participants also completed a questionnaire. Data analysis was done using SPSS version 20.

**Result:**

A total of 166 pregnant women participated in this study. The mean age of the participants was 29 ± 4.3 years. The HIV prevalence was highest in the 25–29 years category. This was also the modal age category. Majority of the women were multiparous. The prevalence of HIV infection was 12 %. The sensitivity, specificity, negative and positive predictive value of serial rapid HIV testing was 95, 100, 99.3 and 100 % respectively.

**Conclusion:**

The sensitivity of the serial rapid test algorithm was high but still lower than the WHO recommended 99 % and above. The 100 % specificity and positive predictive value makes it a good diagnostic test strategy. There is need for regular review of HIV test kits and policy.

## Background

It has been estimated that sub-Sahara Africa contributes 22.4 million of the 32.4 million cases of HIV infection worldwide [1]. This represents 67 % of HIV infection. More worrisome is the fact that HIV in this region is wearing a woman’s face with 61 % of HIV infections in this region occurring in women [1]. This has a detrimental implication for mother to child transmission of HIV infection. Overall, it has been estimated that 60 million people have been infected with HIV since the beginning of the epidemic and 25 million people have died of HIV-related causes [2].

Nigeria’s HIV infection is peculiar because of the country’s population. It has been estimated that Nigeria has one of the highest number of people living with HIV infection [3]. The annual HIV exposed births in Nigeria in 2009 was 56,681 [4]. More than 90 % of paediatrics HIV infections are as a result of mother to child transmission [5]. Diagnosis is also more challenging because of presence of HIV-2 in the region.

HIV prevention and treatment hinges on the availability of accurate and feasible diagnostic tests. HIV counseling and testing is the gateway for HIV prevention, care, treatment and support of interventions. Among pregnant women, screening has proven to be more effective than risk-based approach for detecting unsuspecting maternal HIV infection [6]. Screening for HIV infections were previously done using conventional ELISA and confirmed with western blot testing until the evolution rapid HIV testing kits. Both parallel and serial rapid test algorithm has been used for screening and diagnosis of HIV infection in Nigeria.

The routine use of conventional ELISA was abandoned because it is technically demanding, and requires sophisticated, regularly maintained equipment and constant supply of electricity. The ELISA kits use both natural and recombinant antigens. They are updated continuously to increase their sensitivity to newly discovered species like group O viruses [7]. The fourth generation ELISA also detects the presence of p24 antigen thereby improving the sensitivity.

Rapid tests for the detection of antibodies to HIV have revolutionized the diagnosis and management of patients with HIV infection. It allows timely point of care, provision of results and do not require the laboratory facilities needed for conventional ELISA and western blot testing [8–11]. Rapid HIV tests have been widely used for voluntary HIV counseling and testing, antenatal surveillance, and population screening [10, 11]. These tests are simple to use and cheaper than laboratory based HIV antibody tests [12]. Introduction of rapid HIV tests has contributed in the scaling up of HIV-testing programme and enabled diagnosis of HIV in persons who might not have had their infections diagnosed otherwise [13–17]. This is more pertinent in sub-Sahara Africa with very high disease burden and weak health sector.

The sensitivity of many rapid test kits is comparable to the conventional enzyme immunoassays [15–18]. Eller et al. reported a sensitivity of 98.6 % and specificity of 99 % in Uganda [16]. This agrees with the result of several studies [18–20]. These results met the WHO criteria of 98 % sensitivity and 99 % specificity. Rapid HIV tests have been developed for on-site real time testing and quality of testing is dependent on user and test kits. High false positive rates have been observed with some test kits [21–25].

Studies have reported poor positive predictive value of three rapid tests if weak bands are interpreted as positive according to manufacturers’ recommendation [23, 26]. It has been postulated that weak positive bands may reflect cross-reactions with other infections [23].

Various HIV testing algorithms were introduced to improve the accuracy of testing. These testing algorithms involve a combination of tests used either in sequence or parallel, designed to achieve predictive values close to 100 %. These include the parallel testing and serial testing. Parallel screening is the use of two tests simultaneously, each test being a check on the other, and can be considered to create efficiency. Serial testing involve the use of rapid test kit with sensitivity close to 100 %. Second test is done only if the initial test is positive. The parallel approach is expensive and in lower prevalence settings, may lead to the use of longer numbers of tests than the use of a serial strategy. Currently serial rapid testing is the option in Nigeria based WHO recommendation.

Despite the improvement to access and wide scale implementation, quality assurance mechanism is very important to validate the accuracy as stated by the manufacturers. In addition, it will also detect post market problems like batch variations, storage problems and fake products.

High false positive rates have an adverse effect on the health system and individuals. The individuals will suffer stigma and discrimination, with attendant psychological and social consequences. She may also be prone to domestic violence and exposed to the unnecessary and potentially toxic medical treatment. This will lead to increase in antiretroviral drugs budget and will put a heavy strain on the health sector especially in developing countries. In addition, a HIV negative person will be meant to live with the stigma associated with the disease with attendant psychological and social consequences. False negative results have a great implication for mother to child transmission of HIV infection. These women are denied the benefit of antiretroviral therapy in reduction of mother to child transmission.

Studies have shown reduced ability of rapid test kits in detecting some strains of HIV-1 and HIV-2 antibodies when compared with conventional ELISA [26–29]. More worrisome is the observation that some rapid test kits used in different algorithm detect the same type of antigens contrary to WHO recommendations [30].

Rapid point of care HIV tests has created new challenges and opportunities for service providers, policy makers and researchers concerning broad scale identification of HIV seropositive persons. The major challenge is to identify most suitable assays for a given circumstance without compromising the test results. It is essential to establish quality assurance programs so that benefits of rapid point of care HIV test will be optimized. This will help to detect batch to batch differences, storage and other post market problems that may hinder the accuracy of the test kits.

Periodic evaluations of these kits will help in making sure that the kits meet the minimum standard. This study evaluated the accuracy of the serial rapid test algorithm by determining the sensitivity, specificity, negative and positive predictive values of the serial rapid HIV test algorithm among booked antenatal women in Nnewi, South East Nigeria, which is one of the centres of excellence for prevention of mother to child transmission of HIV infection in Nigeria.

## Subjects, materials and methods

### Study design

This was a descriptive comparative study conducted among pregnant women. The sensitivity, specificity, negative and positive predictive value of rapid HIV kits (Determine and UNi-Gold) using the serial testing algorithm was determined. We used conventional ELISA as the gold standard for screening and Western blot as the Gold standard for confirmation of all positive and discordant results. A total of 166 pregnant women participated in this study. This study was approved by the ethics committee of Nnamdi Azikiwe University Teaching Hospital, Nnewi, Anambra state, Nigeria.

### Study area

The study was conducted at Nnamdi Azikiwe University Teaching Hospital (NAUTH) Nnewi, between January 2012 and April 2012. NAUTH is one of the pioneer PMTCT referral centre of excellence in Nigeria.

### Study population

This comprised of pregnant women attending antenatal clinic in NAUTH, Nnewi within the study period.

### Inclusion criteria

1. Pregnant women presenting at antenatal clinic in NAUTH.
2. Pregnant women that gave informed consent.

### Exclusion criteria

1. Non pregnant women
2. Pregnant women previously confirmed HIV seropositive.
3. All pregnant women that withheld consent for the study.

### Measure of outcome

The main outcome measure was determination of sensitivity, specificity, negative and positive predictive value of serial rapid test algorithm using Determine-1/2 and Uni-Gold rapid test kits.

### Laboratory procedures

Pre-test counseling was carried out after completion of the questionnaire. The researcher or any of the research assistant performed a venipuncture to collect 5 ml of blood. Part of the blood sample was emptied into an EDTA bottle that was appropriately coded to match the subject’s questionnaire for easy identification. This was centrifuged and the serum stored for conventional ELISA batch testing (Bio Rad, France) and western blot testing (NEW LAV BLOT 1 by Bio Rad, France) where applicable. The samples for western blot were analysed at the central public laboratory Yaba, Lagos. The remaining sample was handed to Institute of human virology of Nigeria (IHVN) scientist for HIV screening using Determine HIV-1/2^®^(Alere Japan) and UniGold HIV rapid test kits (Trinity Biotech Ireland) where applicable. The first test was done using Determine HIV rapid test kit. Those that tested negative were declared negative. Those that tested positive for the first test were retested using Unigold HIV rapid test kit. Concordant or discordant positive samples were sent for confirmatory test with western blot. These women were told that further test will be done to confirm the result. They were given immediate appointment in the antenatal clinic. The subject that had false result was contacted through the antenatal clinic. She was enrolled into the PMTCT programme after counseling. All the clients, regardless of the result received routine counseling on risk reduction practices. The folder was tagged to avoid double recruitment of the subject. The manufacturer’s instructions were strictly adhered to in performing the tests. The rapid tests and ELISA test were done at Nnamdi Azikiwe University Teaching Hospital Nnewi while the western blot test was carried out at the central public health laboratory in Yaba, Lagos, Nigeria using NEW LAV BLOT 1 (BIO RAD France). The cold chain was maintained in transporting the samples from Nnewi to Lagos.

The flow chart is shown in the table below 

*SRT* serial rapid test algorithm, *EIA* conventional ELISA, *WB* western blot.

### Disclosure of result

The client was given a negative result in an individualized posttest counseling session. The confirmed positive results were disclosed in an individualized posttest counseling session in the follow up appointment. The discussion on the disclosure of the result was made as well as partner and family testing. The patient was enrolled in the hospital PMTCT services and social support services. False negative result was defined as a sample that gave a negative result with the first rapid test and classified as negative according to the serial test algorithm but was confirmed positive with western blot. False positive result was defined as a sample that gave a positive test result with two independent rapid tests, and classified as positive according to the WHO two-test algorithm, which was not confirmed by western blot testing.

### Statistical analysis

Statistical analysis of the result was done using SPSS version 20.0. The level of statistical significance was set at p value 0.05 providing 95 % confidence interval.

### Limitations of the study

The constraint of financial resources limited the sample size because the researchers bore all the expenses incurred in the course of the study.

## Ethical consideration

Written informed consent was obtained from the subjects that participated in the study and the study was performed in accordance with the Declaration of Helsinki. Ethical clearance was by the ethics committee of Nnamdi Azikiwe University Teaching Hospital, Nnewi, Anambra state Nigeria before the commencement of the study.

## Results

One hundred and sixty-six pregnant women participated in this study. The mean age of the women was 29 years ± 4.3. Majority of the women were in the age category of 25–29 years 9(47.4 %). Two were below 20 years while only one was above 40 years. Only one woman was 40 years.

A greater percentage (96.4 %) was currently married. Two women each (1.2 %) were single, divorced or widowed respectively. Twenty women were confirmed HIV positive with western blot giving a prevalence of 12.04 %. None of the positive women was below 20 years. The highest HIV prevalence was documented in the 25–29 age category.

The evaluation of the serial rapid test algorithm showed that 19 patients tested positive. There was one false negative but no false positive (sensitivity of 95 % and specificity of 100 %). Twenty-seven tested positive to the conventional ELISA test and western blot confirmed only 20 of the women positive for HIV. There were 7 false positive and no false negative (sensitivity of 100 % and specificity of 95.3 %) for the conventional ELISA. Table 1 shows the distribution of disease state and age group while Table 2 shows rapid test and ELISA test concordance.

**Table 1:** Age and confirmed result

Age range	Negative N %		Positive N %		Total N %	
<20	2	1.2	0	0.0	2	1.2
20–24	19	11.4	2	2.4	21	12.7
25–29	55	33.1	9	5.4	64	38.6
30–34	50	30.1	6	3.6	56	33.7
35–39	20	12.0	2	1.2	22	13.2
≥40	0	0.0	1	0.6	1	0.6
Total	146	88	20	12.0	166	100

**Table 2:** Rapid HIV and ELISA concordance

Concordance	Frequency	Percentage
Negative concordance	139	83.7
Positive concordance	19	11.5
RAPID test positive, ELISA negative	0	0
RAPID test negative, ELISA positive	8	4.8

Sensitivity of the serial rapid test was 95 % while the specificity was 100 %. The positive predictive value was 100 %. The evaluation of serial rapid HIV and ELISA test results are shown in Table 3. Table 4 shows Rapid Test, ElISA and Western blot concordance.

**Table 3:** Accuracy of serial rapid test algorithm

Test result	Disease present	Disease absent	Total
Positive	19	0	19
Negative	1	146	147
Total	20	146	166

Sensitivity = 95 %, specificity = 100 %, negative predictive value = 99.3 %, positive predictive value = 100 %

**Table 4:** Rapid test, ELISA and western blot concordance

Cohort	Number
SRTA positive, EIA positive, WB positive	19
SRTA negative, EIA positive, WB Positive	1
SRTA negative, EIA positive WB negative	7

*SRTA* serial rapid test algorithm, *EIA* conventional ELISA, *WB* western blot

## Discussion

The mean age of the women in this study was 29 years ± 4.3 and the modal age category was 25–29 years. This age category also has the highest number of infected women. This finding agrees with the highest age specific prevalence of HIV infection among women in a national survey in Nigeria [31]. This has a great implication on mother to child transmission of HIV infection. This is because these women are at the peak of their reproductive life. It may also affect the overall HIV prevalence rate bearing in mind the high fertility rate in Nigeria and the total population of the country. The prevalence of HIV infection among pregnant women in this study was 12 %. This is high but comparable figure 10.5 % recorded in a previous in Nnewi [32]. The high prevalence rate may be because the hospital is a referral centre for the management of HIV positive patients in the region.

In this study, the sensitivity of serial rapid test algorithm was 95 %. This is high but lower than the WHO recommended sensitivity of rapid test of ≥99 % [33]. Most of the published works on accuracy of rapid test in HIV diagnosis in Nigeria evaluated each kit separately. Nkwocha recorded a lower sensitivity of 86.6 % for Determine rapid kits which is lower than the observation in our study [34]. The difference may be due to the use of a serial rapid test algorithm in our study. Using an established algorithm as recommended by WHO in the screening of HIV improves the accuracy of the study (i.e. the sensitivity, specificity, positive predictive value and negative predictive value).

The specificity and positive predictive value of serial rapid test algorithm in this study was 100 %. Several studies have recorded specificity and positive predictive value of 98 % and above which meets the WHO recommendation [16–18]. This is good news considering the fact that in low resource countries, rapid HIV kits are used for both screening and confirmation of diagnosis. Well-established algorithms have contributed immensely to the scaling up of HIV testing, diagnosis, prevention and treatment.

A case of false negative result recorded in this study underscores the potential for false result when using rapid test algorithm for screening and diagnosis of HIV infection in pregnant women. Some studies have also observed false results with HIV test algorithm which underscores the need for periodic evaluation of different HIV testing strategies [35–37]. Klarkowski et al. in a multicenter and multi country study observed variations in accuracy of testing between regions and centres in Ethopia and Myanner [37]. Bavewo et al. also noted high false positive result with serial rapid HIV test algorithm [38].

Our study did not evaluate different points of flaws in testing. Another multicenter study that will include primary, secondary and tertiary health centres will be needed to determine the various points of flaws in the testing. These will help to maintain the accuracy of testing and good quality assurance.

The high rate of false positive result recorded with conventional ELISA calls for further study. Causes of false positive results include cross reactivity or non-specific reactivity. Some authors have also propounded that the false positive results may be due to some strains of HIV-2 and HIV-1 group O whose antigens are not easily detected [35, 36]. Further studies will be required to prove or refute this submission.

This study confirms the known fact that rapid HIV testing offers an enormous opportunity for HIV screening and diagnosis and linkages to other HIV services aimed at reducing the HIV/AIDS scourge. It is more pertinent in developing countries where it can be used for screening and diagnosis. However, there is need for local and national quality assurance program to make sure that high standards are always maintained. This can be achieved by periodic evaluation of rapid HIV test kits and different test algorithm. Institution-based and national periodic evaluation is very important in maintaining high accuracy of testing.

## Conclusion

Despite the high accuracy of serial rapid test algorithm, there is still room for improvement of testing because of the potential for false results as recorded in this study and other studies. There should be a mechanism for periodic evaluation at various levels of testing ranging from primary, secondary and tertiary health institutions. This will help to ascertain point of testing accuracy and identify some of the flaws associated with testing. We also propose that conventional ELISA test may be used in tertiary centres as part of the testing strategies to reduce false negative results. There is need for sustained funding of research in this area by international, regional and local institutions. Retesting of pregnant women in labour will also help to improve dictation rate and better patients’ management.

## Abbreviations

NAUTH: Nnamdi Azikiwe University Teaching Hospital
PMTCT: prevention of mother to child transmission of HIV
IHVN: Institute of human virology of Nigeria
WHO: World Health Organisation
HIV: human immunodeficiency virus
